# A multicenter randomized controlled trial evaluating the effect of the use of an anti‐adhesion barrier for diverting ileostomy on the multidimensional workload in minimally invasive surgery for rectal cancer (YCOG 2005: The ADOBARRIER study)

**DOI:** 10.1002/ags3.70009

**Published:** 2025-03-06

**Authors:** Emi Ota, Jun Watanabe, Yusuke Suwa, Masakatsu Numata, Hirokazu Suwa, Hiroki Ohya, Kazuya Nakagawa, Mayumi Ozawa, Itaru Endo

**Affiliations:** ^1^ Department of Surgery Yokosuka Kyosai Hospital Yokosuka Japan; ^2^ Department of Colorectal Surgery Kansai Medical University Hirakata Japan; ^3^ Department of Surgery, Gastroenterological Center Yokohama City University Medical Center Yokohama Japan; ^4^ Department of Gastroenterological Surgery Yokohama City University Graduate School of Medicine Yokohama Japan

**Keywords:** anti‐adhesion barrier, diverting ileostomy, ileostomy closure, rectal cancer, SURG‐TLX

## Abstract

**Aim:**

The purpose of this study was to assess whether the use of spray‐type anti‐adhesion material during diverting ileostomy construction could reduce the surgeon's multidimensional workload, the degree of adhesion, and the operation time in ileostomy closure.

**Methods:**

Patients diagnosed with rectal cancer, who were scheduled for laparoscopic or robotic rectal surgery followed by diverting ileostomy, were single‐blindly (patient‐blind), randomly assigned to either the AdSpray™ arm or the control arm. The primary endpoint was the multidimensional workload of the ileostomy closure operator (SURG‐TLX value).

**Results:**

Between January 2020 and December 2022, 126 patients were enrolled. Five patients were excluded and a total of 121 patients (control arm, *n* = 60; AdSpray™ arm, *n* = 61) were analyzed. The baseline factors were well balanced between the two arms. Regarding SURG‐TLX in ileostomy closure, operators in the AdSpray™ arm required a significantly lower overall workload than those in the control arm (AdSpray™ arm, 28.1; control arm, 58.9; *p* < 0.001). Mental, physical, and temporal demands, task complexity, situation stress, and distractions were significantly lower in the AdSpray™ arm (*p* < 0.001). Operative time was significantly shorter in the AdSpray™ arm (AdSpray™ arm, 58 min; control arm, 65 min; *p* = 0.040). The degree of adhesion (*p* < 0.001) and extent of intra‐abdominal adhesions (*p* < 0.001) in ileostomy closure were significantly lower in the AdSpray™ arm.

**Conclusions:**

The use of spray‐type anti‐adhesion material was associated with a significantly lower SURG‐TLX value, lower incidence of adhesion, less severe adhesion, and shorter operative time.

## Graphical abstract

This multicenter, single‐blind, randomized, controlled trial was conducted to evaluate the multidimensional workload of the first operator during ileostomy closure using the SURG‐TLX between groups with and without the use of the spray‐type anti‐adhesion material during diverting ileostomy construction. The use of spray‐type anti‐adhesion material was associated with a significantly lower SURG‐TLX value, lower incidence of adhesion, less severe adhesion, and shorter operative time.

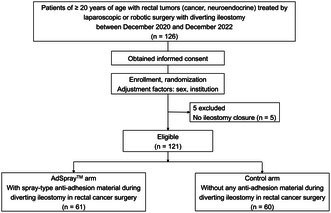

## 1 INTRODUCTION

It occurs after all types of surgery and has been shown to prolong the operative time, increase technical difficulty, and cause complications.^1^, ^2^, ^3^, ^4^ The incidence rate of peritoneal adhesions has been reported to be 55%–95%.^5^, ^6^ Laparoscopic surgery is to open surgery in the prevention of intra‐abdominal adhesion, but even with laparoscopic surgery, small bowel obstruction still occurs in 7%–8% of patients.^7^, ^8^, ^9^ Of these, intestinal surgery is associated with the highest risk of adhesion, and the colon and rectum have the highest risk of peritoneal adhesion in the first year after surgery.^10^, ^11^ However, there is no consensus on how to prevent adhesions, and no guidelines have been developed to prevent adhesions in clinical practice.^12^, ^13^

Recent advances in surgical techniques, including robotic surgery and preoperative chemoradiotherapy, have made it possible to preserve the lower rectum and anal sphincter. This has led to an increase in the number of diverting ileostomies. The problem with diverting ileostomy is that a second operation is required and adhesions from the initial operation for primary site resection may be an obstacle during ileostomy closure. Strong adhesion between the ileostomy and the abdominal wall or cavity leads to intraoperative bowel injury and prolongs the operative time of ileostomy closure, causing great stress to the surgeon. However, there have been few reports on the effects of anti‐adhesion materials on ileostomy closure.^14^, ^15^, ^16^, ^17^

To reduce postoperative adhesions, Terumo Corporation (Tokyo, Japan) developed a spray‐type anti‐adhesion barrier system (AdSpray™) that can be easily applied to various surgical sites, including laparoscopic or robotic surgery. AdSpray™ is a bioabsorbable hydrogel consisting of N‐hydroxysuccinimide‐modified carboxymethyl dextrin, which can be applied as a spray using this kit. Suto et al.^18^ reported that the use of AdSpray™ in patients undergoing diverting ileostomy for primary rectal cancer significantly reduced adhesions under the midline wound during ileostomy closure (50% vs. 90%). With AdSpray™, anti‐adhesion materials can be freely sprayed between the ileostomy and abdominal wall and between the ileostomy and peritoneum in the abdominal cavity. By reducing the adhesion between the ileostomy and the abdominal wall and the adhesion in the abdominal cavity, peeling of the ileostomy circumference during ileostomy closure can be managed, thereby reducing the physical and mental stress on the surgeon in comparison to the conventional procedure (without anti‐adhesive materials). In addition, the operative time may be reduced by reducing the time required for adhesiolysis.

The aim of the present study was to evaluate whether spraying an anti‐adhesion material during the creation of a diverting ileostomy could reduce the multidimensional workload of the first operator of ileostomy closure, the operation time, and the degree of adhesion in ileostomy closure. We evaluated the surgeon's stress by using SURG‐TLX. The SURG‐TLX is a validated surgery‐specific questionnaire that takes several possible stressors in an operating room into account. The workload in surgeons has different dimensions, such as mental demands, physical demands, temporal demands, task complexity, situational stress and distraction, all of which can increase the surgeon's stress during work.^19^, ^20^

## 2 METHODS

### 2.1 Patients

This multicenter, single‐blind (patient‐blind), randomized, controlled trial was conducted, as has been previously described,^21^ to evaluate the multidimensional workload of the first operator during ileostomy closure using the surgery task load index (SURG‐TLX) between groups with and without the use of the spray‐type anti‐adhesion material during diverting ileostomy construction as the primary endpoint; operative time, amount of intraoperative blood loss, degree of adhesions, and extent of intra‐abdominal adhesions at ileostomy closure as the secondary endpoints. The study was approved by the Ethical Advisory Committee of Yokohama City University Graduate School of Medicine and the Institutional Review Board of each participating hospital before the initiation of the study (No. CRB3180007), and was conducted in accordance with the principles of the Declaration of Helsinki. Informed consent was obtained from all patients. This trial was registered in the Japan Registry of Clinical Trials (jRCT) in October 2020 as jRCTs032200155.

### 2.2 Patient selection

The inclusion criteria were as follows: (1) age ≥ 20 years (legal age of adulthood in Japan at the time of study enrollment); (2) a preoperative diagnosis of primary rectal cancer (including adenocarcinoma, squamous cell carcinoma, adenosquamous carcinoma, and neuroendocrine tumor); (3) rectal cancer surgery followed by construction of a diverting ileostomy; (4) ileostomy closure within 6–48 weeks after primary surgery; and (5) written consent for study participation. The exclusion criteria were as follows: (1) creation of a diverting colostomy; (2) open surgery; (3) judged to be unable to fully understand the study content; (4) pregnant patients who might become pregnant or who wished to become pregnant during treatment; and (5) patients deemed ineligible for participation in this study by the attending physician.

### 2.3 Surgeon's criteria

There were no surgeon's criteria for the primary surgery. For ileostomy closure, the criteria was that the surgeon must have completed more than five stoma closure procedures as an attending surgeon.

### 2.4 Randomization

Randomization and data handling were performed using an electronic data capturing system (Gravity Data Management System; [Medical Edge, Tokyo, Japan]). After confirming the inclusion and exclusion criteria and obtaining written informed consent, the patients were randomly assigned (1:1) to the AdSpray™ arm (spray‐type anti‐adhesion material was used during surgery) or the control arm (no anti‐adhesion material‐was used). Randomization was performed by the minimization method using the institution and sex, as well as adjustment factors to prevent significant bias from occurring with these factors. The investigators at the participating institutions were blinded to the randomization algorithm. The investigator surgeons were informed of the treatment allocation via the Internet and performed the procedure. The patients were blinded to the group assignment.

### 2.5 Surgical procedure

**FIGURE 1:**
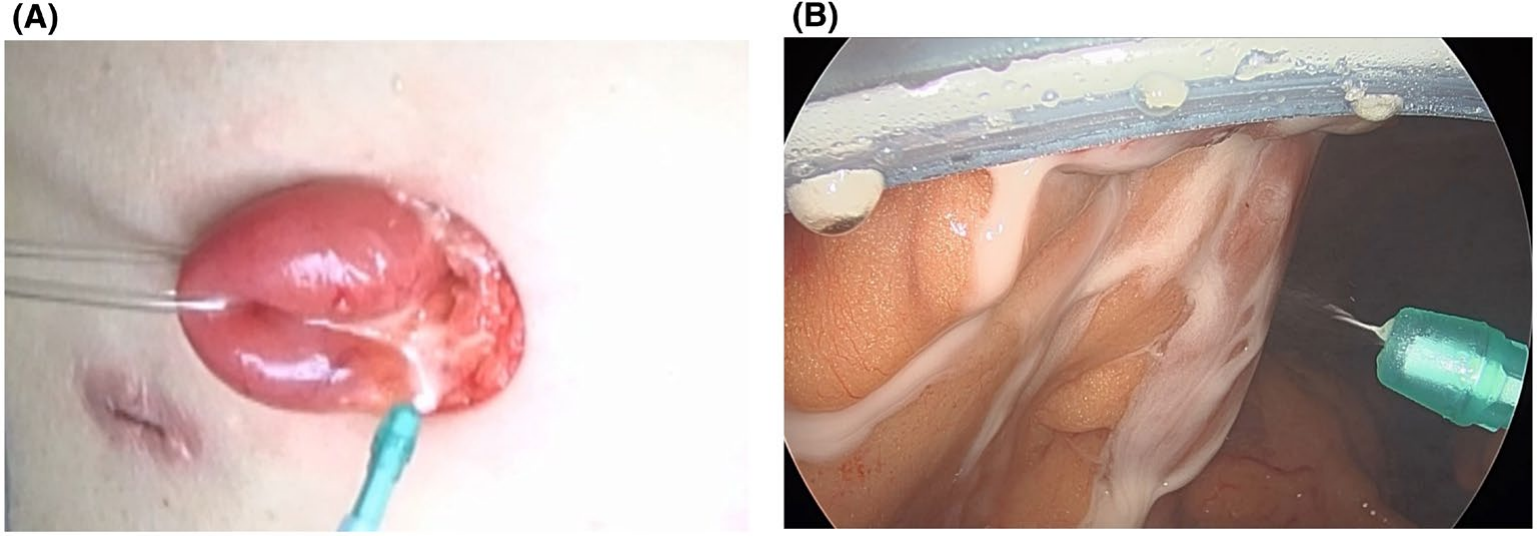
(A) Use of spray‐type anti‐adhesion material between the ileostomy and abdominal wall. (B) Use of spray‐type anti‐adhesion material between the ileostomy and peritoneum.

In the control group, diverting ileostomy was performed without any anti‐adhesion material (and the use of any anti‐adhesion material outside the ileostomy site was also unacceptable). In the study group, spray‐type anti‐adhesion material was sprayed between the ileostomy and the abdominal wall (Figure 1A) and between the ileostomy and peritoneum (Figure 1B) during the construction of the diverting ileostomy. Only AdSpray™ was used as an anti‐adhesion material in the present study, and the use of other anti‐adhesion materials was prohibited. Surgeons who had performed fewer than five stoma closures as attending surgeons were excluded.

### 2.6 Endpoints

The primary endpoint was the multidimensional workload of the first ileostomy closure operator, which was assessed using the total score obtained from the SURG‐TLX questionnaire. Law et al.^22^ compared the multidimensional workloads evaluated by NASA‐TLX stratified by the surgical approach. SURG‐TLX is a modified version of NASA‐TLX, with “Effort” changed to “Tak complexity,” “Performance” to “Situational stress,” and “Frustration” to “Distractions,” a scoring system developed for surgeons.^20^ The surgeon performing ileostomy closure was asked to complete the SURG‐TLX questionnaire immediately after surgery for ileostomy closure. The SURG‐TLX questionnaire consists of six items (mental demands, physical demands, temporal demands, task complexity, situational stress, and distractions). Each item was scored with a maximum of 20 points for a total of 120 points. The secondary endpoints were operative time for ileostomy closure, intraoperative blood loss volume for ileostomy closure, assessment of adhesions during ileostomy closure, and status of intestinal detachment (degree of intestinal injury) during ileostomy closure. Assessment of adhesions during ileostomy closure were (i) presence or absence of adhesions in the abdominal cavity, defined as no adhesions (0) or some adhesions (1); (ii) severity score of adhesion with the abdominal wall, defined as no adhesions (0); thin film, avascular (1); moderate thickness, limited area of vascularization (2); or thick, vascularized (3); (iii) assessment of the extent of peritoneal adhesions, defined as no adhesions (0), covering ≤50% of the total area/length of the target area (1); or covering >50% of the total area/length of the target area (2). The status of intestinal detachment during ileostomy closure was defined as no intestinal injury (0), a small area of serosal injury (1), extensive serosal injury (2), and injury to all layers of the intestinal tract (3). In addition, intraoperative complications during ileostomy at the primary operation, postoperative complications within 30 days after the primary operation, intraoperative complications during ileostomy closure, and postoperative complications within 30 days after ileostomy closure will also be evaluated for safety.

### 2.7 Statistical analyses

All study subjects enrolled in this trial who underwent laparoscopic rectal surgery after randomization and for whom efficacy data were available were considered part of the full analysis set (FAS). However, subjects for whom baseline data could not be obtained or those with severe violations of the research implementation plan (non‐acquisition of content, registration outside the contract period, etc.) were excluded. For patient background factors, data are presented as the median and interquartile range. For the primary endpoint, the obtained data are presented as the mean ± SD, and were statistically evaluated using Student's *t*‐test. If the obtained data did not show a normal distribution, the values were presented as the median and interquartile range (IQR), and statistically evaluated using the Mann–Whitney *U* test. Two‐sided *p* values of <0.05 were considered statistically significant. For secondary endpoints, Pearson's chi‐square test was used to test the superiority of the two groups in the presence or absence of adhesions. Adhesion severity and adhesion range scores were tested for superiority using Fisher's exact test. Statistical analyses were performed using the IBM SSPS Statistics software program, ver. 27.0 (IBM Corporation, Somers, NY, USA).

## 3 RESULTS

### 3.1 Patient characteristics

**FIGURE 2:**
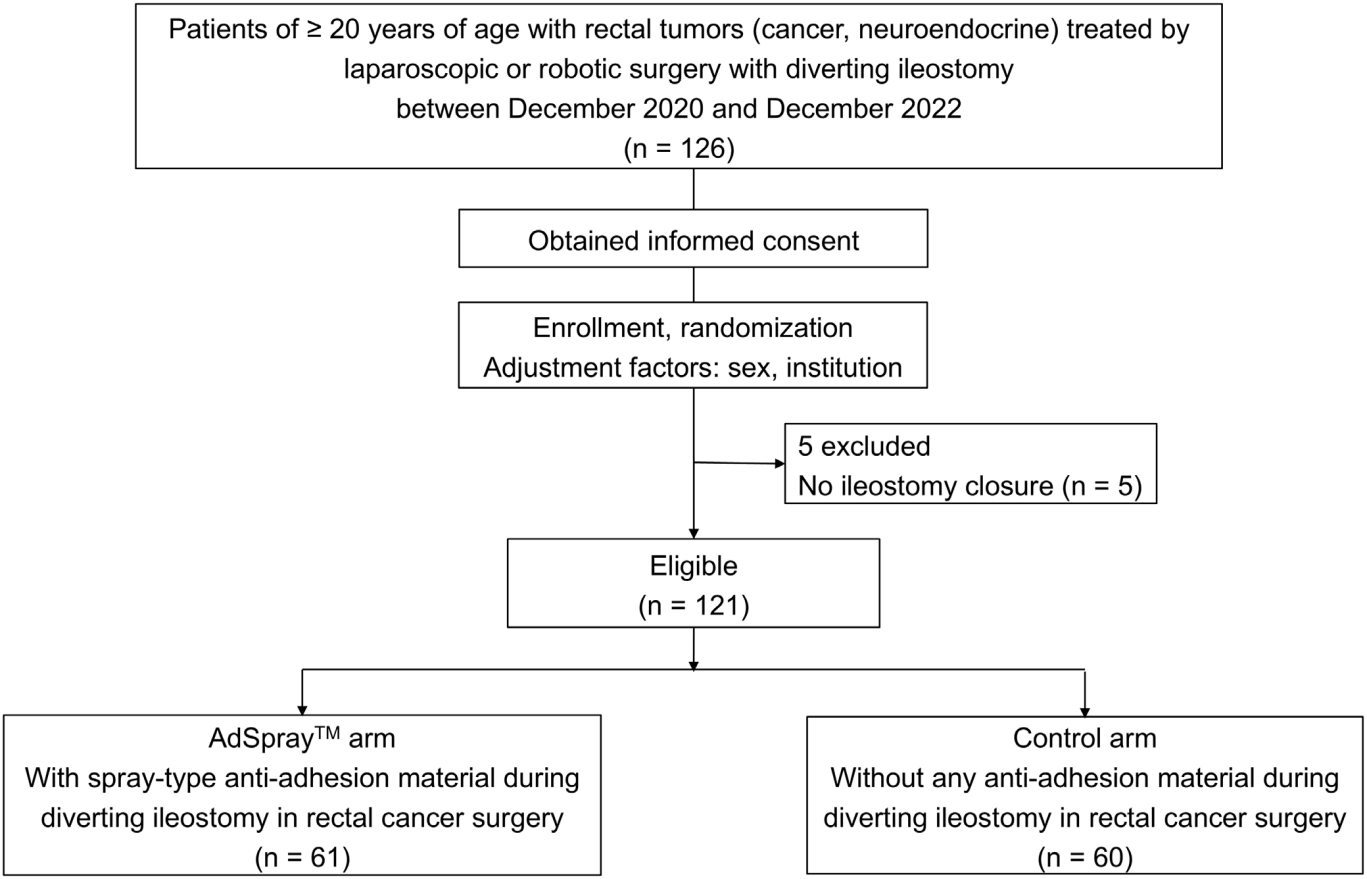
Flowchart of patient selection.

Between December 2020 and December 2022, 126 eligible patients were registered from three institutions of the Yokohama Clinical Oncology Group (YCOG) (Yokosuka Kyosai Hospital, Yokohama City University Medical Center, and Yokohama City University Hospital). Five patients were excluded due to the absence of ileostomy closure. Consequently, 121 patients were subjected to FAS analysis (AdSpray™ arm, *n* = 61; control arm, *n* = 60) (Figure 2). The clinical characteristics and surgical outcomes of the primary surgery in the two groups are shown in Table 1. Patient background, age, sex, performance status (PS), body mass index (BMI), American Society of Anesthesiologists Physical Status Classification System (ASA‐PS) score, comorbidities, and history of abdominal surgery were well‐balanced between the two groups. Oncological factors, distance of the tumor from the anal verge, clinical stage, and neoadjuvant therapy were also well‐balanced between the groups. Operative factors, approaches, operative time, blood loss, postoperative complications, and postoperative length of hospital stay were also not different between the groups.

**TABLE 1:** Clinical characteristics and surgical outcomes of the primary surgery.

	AdSpray™ arm (*n* = 61)	Control arm (*n* = 60)	*p* Value
Age, median, years	65 (58–70)	65.5 (56–71)	0.929*
Sex			
Male	38 (62.3%)	40 (66.7%)	0.615
Female	38 (37.7%)	20 (33.3%)	
PS			
0	57 (93.4%)	57 (95.0%)	0.714
1	4 (6.6%)	3 (5.9%)	
BMI, median, kg/m^2^	22.4 (20.0–25.1)	23.6 (21.2–25.7)	0.173*
ASA‐PS score			
1	3 (4.9%)	4 (6.7%)	0.339
2	57 (93.4%)	52 (86.7%)	
3	1 (1.6%)	4 (6.7%)	
c‐Stage			
I	17 (27.9%)	21 (35.0%)	0.397
II	16 (26.2%)	10 (16.7%)	
III	25 (41.0%)	28 (46.7%)	
IV	3 (4.9%)	1 (1.7%)	
Comorbidities			
No	15 (24.6%)	22 (36.7%)	0.149
Yes	46 (75.4%)	38 (63.3%)	
History of abdominal surgery			
No	43 (70.5%)	45 (75.0%)	0.578
Yes	18 (29.5%)	15 (25.0%)	
Neoadjuvant therapy			
No	51 (83.6%)	50 (83.3%)	0.732
Neoadjuvant chemotherapy	1 (1.6%)	1 (1.7%)	
Chemoradiotherapy	1 (1.6%)	3 (5.0%)	
Total neoadjuvant therapy	8 (13.1%)	6 (10.0%)	
Approaches			
Conventional laparoscopic surgery	7 (11.5%)	5 (8.3%)	0.519
Robotic‐assisted laparoscopic surgery	47 (77.0%)	44 (73.3%)	
Trans‐anal total mesorectal excision	7 (11.5%)	11 (18.3%)	
Operative time, min	270 (196–347)	272 (207–373)	0.786*
Blood loss, mL	5 (0–46)	12 (3.8–42.3)	0.238*
Distance of tumor from anal verge, cm	5.7 (4.0–8.0)	5.8 (4–7.1)	0.927*
Postoperative complications (primary surgery)			
No	46 (75.4%)	41 (68.3%)	0.387
Yes	15 (24.6%)	19 (31.7%)	
Anastomotic leakage	1 (1.6%)	0 (0%)	
Bowel obstruction	2 (3.3%)	2 (3.3%)	
Ileus	2 (3.3%)	2 (3.3%)	
Surgical site infection	0 (0%)	2 (3.3%)	
Others	11 (18%)	13 (21.7%)	
Postoperative length of stay, days	12 (10–14)	11 (10–14)	0.084*

*Note*: Values in parentheses are percentages, unless indicated otherwise; values are median (interquartile range: 25–75th percentile). Categorical variables were tested using the Pearson's chi‐square test.

Abbreviations: ASA‐PS, American Society of Anesthesiologists Physical Status Classification System; BMI, body mass index; PS, performance status; SD, standard deviation.

* Mann–Whitney *U* test.

### 3.2 Efficacy

The mean SURG‐TLX value was 28.1 in the AdSpray™ arm and 58.9 in the control arm (*p* < 0.001). The mental demand (4.8 vs. 10.39, *p* < 0.001), physical demand (4.3 vs. 10.4, *p* < 0.001), temporal demand (5.3 vs. 9.9, *p* < 0.001), task complexity (4.7 vs. 10.6, *p* < 0.001), situational stress (4.9 vs. 10.2, *p* < 0.001), and distractions (4.0 vs. 8.6, *p* < 0.001) were significantly lower in the AdSpray™ arm, as shown in Table 2.

**TABLE 2:** Multidimensional workload of the first operator during ileostomy closure.

	AdSpray™ arm (*n* = 61)	Control arm (*n* = 60)	*p* Value
SURG TLX (overall workload)	28.1 (±15.6)	58.9 (±24.8)	<0.001
Mental demand, average	4.8 (±2.7)	10.4 (±4.6)	<0.001
Physical demand, average	4.3 (±2.7)	9.3 (±4.1)	<0.001
Temporal demand, average	5.3 (±3.5)	9.9 (±4.5%)	<0.001
Task complexity, average	4.7 (±3.3)	10.6 (±4.9)	<0.001
Situation stress, average	4.9 (±3.1)	10.2 (±4.6)	<0.001
Distractions, average	4.0 (±2.5)	8.6 (±3.6)	<0.001

*Note*: The data are presented as the mean (± standard deviation), and were statistically evaluated using Student's *t*‐test.

The evaluation of adhesions during ileostomy closure is shown in Table 3. The incidence of adhesion in the abdominal cavity was 21.3% in the AdSpray™ arm and 50% in the control arm. The incidence of adhesion was significantly lower in the AdSpray™ arm than in the control arm (*p* < 0.001). In terms of the severity of adhesion with the abdominal wall, in the AdSpray™ arm, 62.4% of patients had no adhesions (6.6%) or only thin film avascular adhesions (55.8%) to the abdominal wall. No thick or vascularized adhesions were observed. The severity scores of adhesions with the abdominal wall in the AdSpray™ arm were significantly lower than in the control arm (*p* < 0.001). Regarding the assessment of the range of adhesion in the abdominal cavity, 75.4% of patients in in the AdSpray™ arm had no adhesions and none covered >50% of the total area/length of the target area (*p* < 0.001). The range of adhesion in the abdominal cavity in the AdSpray™ arm was significantly lower than that in the control arm (*p* < 0.001). Regarding the status of intestinal detachment at ileostomy closure, 78.7% of patients in in the AdSpray™ arm had no intestinal injury and only one case of injury to all layers of the intestinal tract. The AdSpray™ arm had a significantly lower incidence of intestinal injury than the control arm (*p* < 0.001).

**TABLE 3:** Evaluation of adhesions during ileostomy closure.

	AdSpray™ arm (*n* = 61)	Control arm (*n* = 60)	*p* Value
Adhesions in the abdominal cavity			
No	48 (78.7%)	30 (50%)	<0.001
Yes	13 (21.3%)	30 (50%)	
Severity score of adhesions with the abdominal wall			
No adhesions	14 (6.6%)	2 (3.3%)	<0.001
Thin film, avascular^a^	34 (55.8%)	22 (36.7%)	
Moderate thickness, Limited range of vascularization^b^	13 (21.3%)	21 (35.0%)	
Thick, vascularized^c^	0 (0%)	15 (25.0%)	
The evaluation of the range of adhesions in the abdominal cavity^d^			
No adhesions	46 (75.4%)	28 (46.7%)	<0.001
Covering ≤50% of the total area/ length of the target area	15 (24.6%)	26 (43.3%)	
Covering >50% of the total area/ length of the target area	0 (0%)	6 (10.0%)	
State of intestinal detachment in ileostomy closure			
No intestinal injury	48 (78.7%)	21 (35.0%)	<0.001
A small range of serosal injury	12 (20.0%)	30 (50.0%)	
Extensive serosal injury	0 (0%)	3 (5.0%)	
Injury to all layers of the intestinal tract	1 (1.6%)	6 (10.0%)	

*Note*: Values in parentheses are percentages. Categorical variables were tested using the Fisher's exact test.

^a^ Separated easily with blunt dissection.

^b^ Separated with blunt or sharp dissection.

^c^ Separated only with sharp dissection.

^d^ The total circumference of the ileostomy is assumed 100%. Adhesion is less than half the circumference of the ileostomy (≤50%) or more than half the circumference (>50%).

The operative and postoperative outcomes of the ileostomy closure are shown in Table 4. The median number of days to ileostomy closure was 105 days in the AdSpray™ arm and 116.5 days in the control arm, with no difference between the groups (*p* = 0.233). The median operative time in the AdSpray™ arm was significantly shorter than that in the control arm (AdSpray™ vs. control: 58 vs. 65 min, *p* = 0.040), and there were no significant differences in blood loss (5 vs. 0 mL, *p* = 0.398). Furthermore, there were no significant differences in the rate of postoperative complications (AdSpray™ vs. control: 8.2% vs. 11.7%, *p* = 0.523) or median length of hospital stay after ileostomy closure (6 days vs. 6 days, *p* = 0.897).

**TABLE 4:** Operative and postoperative outcomes of ileostomy closure.

	AdSpray™ arm (*n* = 61)	Control arm (*n* = 60)	*p* Value
Days to ileostomy closure, days	105 (82–142)	116.5 (90.5–182.5)	0.233*
Operative time, min	58 (43–71)	65 (48.75–78.5)	0.040*
Blood loss, mL	5 (0–5)	0 (0–5)	0.398*
Postoperative complications			
No	56 (91.8%)	53 (88.3%)	0.523
Yes	56 (8.2%)	7 (11.7%)	
Anastomotic leakage	0 (0%)	0 (0%)	
Bowel obstruction	0 (0%)	0 (0%)	
Ileus	2 (3.3%)	1 (1.7%)	
Surgical site infection	1 (1.6%)	0 (0%)	
Others	4 (6.6%)	6 (10%)	
Postoperative length of stay, days	6 (5–7)	6 (5–7)	0.897*

*Note*: Values in parentheses are percentages, unless indicated otherwise; median (interquartile range: 25–75th percentile). Categorical variables were tested using the Pearson's chi‐square test.

* Mann–Whitney *U* test.

## 4 DISCUSSION

The present study focused on the physical and mental stress of the surgeon and adhesion around the ileostomy site during ileostomy closure. The results of this study showed that the use of spray‐type anti‐adhesion material was associated with significantly lower SURG‐TLX values, lower incidence of adhesion, lower severity of adhesions, and shorter operative time. These results suggest that spray‐type anti‐adhesion materials may be beneficial for selected patients undergoing diverting ileostomy. To the best of our knowledge, this is the first report demonstrating that using a spray‐type anti‐adhesion material during ileostomy creation reduces both physical and mental stress on the surgeon during ileostomy closure and minimizes adhesions around the ileostomy site. The spray‐type anti‐adhesion material (AdSpray™) kit has a nozzle of 5 mm in diameter that can be easily inserted into the abdominal cavity through a 5‐mm port, making it easy to use in laparoscopic surgery.^23^ The advantages of the spray‐type anti‐adhesion material include its bendable tip, which allows it to be sprayed on the abdominal wall side; its powder form, which allows the operator to adjust the amount of material that is sprayed; and the ease of spraying on uneven surfaces and in crevices. It is easy to apply in a narrow space, making it easy to use in laparoscopic and robotic surgeries. Although there have been reports that using sodium hyaluronate/carboxymethylcellulose bioresorbable membranes in ileostomy reduces adhesions at the time of ileostomy closure without increasing adverse events and shortens operative time,^14^, ^15^, ^16^, ^17^ no studies have investigated the use of spray‐type anti‐adhesion materials around the ileostomy site. Furthermore, it has several drawbacks as sodium hyaluronate/carboxymethylcellulose bioresorbable membranes is less resilient, brittle, and sticky.^24^ In addition, it is susceptible to induce hemorrhage, poor wound healing, and several other complications.^25^ Unlike other surgeries, diverting ileostomy always requires a second surgery; therefore, it is important to reduce adhesions around the ileostomy site. Because ileostomy closure is performed through a limited wound, dissecting adhesions is challenging. This procedure often requires a longer incision, increases the risk of complications, and adds to the surgeon's stress. Therefore, we assessed both the multidimensional workload of the first ileostomy closure operator and the degree of adhesions. Our results showed that the degree of adhesion matched the surgeon's multidimensional workload, and that the operative time was reduced, which strongly indicates the effectiveness of spray‐type anti‐adhesion materials.

Previous reports have shown that the use of anti‐adhesion materials reduces the incidence of intra‐abdominal adhesions by approximately 50%.^18^, ^26^, ^27^, ^28^, ^29^ Our study also found that the incidence of intra‐abdominal adhesions was reduced by approximately 50%, and that all six indices of the SURG‐TLX (mental demands, physical demands, temporal demands, task complexity, situation stress, and distractions) were reduced by approximately 50% as well. It was considered that the surgeon's multidimensional workload was proportional to adhesions and that as adhesions were reduced, the surgeon's multidimensional workload was reduced in the same proportion.

The safety of spray‐type anti‐adhesion materials has been demonstrated in the ADBEE study and previous clinical trials.^18^, ^30^ Our study also showed no increase in anastomotic leakage or surgical site infection after primary surgery, which has been a concern associated with the spray‐type anti‐adhesion material. In addition, there was no mucocutaneous separation or falling of the ileostomy, which has been a concern because of the spread of anti‐adhesion material around the ileostomy site.

Chandel et al. reported that difficulties during reoperations, rather than small bowel obstructions, account for most adhesion‐related morbidities. For example, iatrogenic bowel injury, a serious adverse event, occurs in 40% of the surgical procedures when adhesiolysis takes over 1 h.^31^ In this study, there were no significant differences in other complications after ileostomy closure between groups. Because this study was a clinical trial, expert colorectal specialists were always required to participate in ileostomy closure at all three institutions. Additionally, the surgeons were required to have experienced at least five ileostomy closures. However, in clinical practice, non‐colorectal surgeons often participate in ileostomy closure, and moreover, ileostomy closure is often performed by young physicians who have just become surgeons. In such a population, there may be differences in postoperative complications between groups that use anti‐adhesion materials and those that do not. Similarly, there was only a small difference in operative time, which might also be due to the fact that the surgeon was a skilled surgeon. Furthermore, the difference in operative time was only 7 minutes in absolute value, but considering that the median operative time for ileostomy closure was about 1 hour, this represents a reduction of approximately 10% of the operative time.

The strength of this study is its randomized design and the evaluation of both the surgeon's multidimensional workload and adhesions, which may provide more reliable results regarding the effectiveness of the use of spray‐type anti‐adhesion materials in reducing intra‐abdominal adhesions. However, our study had several limitations. First, this was a single‐blinded, randomized controlled trial, so it was not possible to blind the surgeons, and there was a possibility of observational bias because the surgeons were aware of the study group assignments. The Certified Review Board directed that whether or not AdSpray™ was used in the primary surgery be noted in the operative record, so surgeon blinding could not be implemented in this study. Second, there was also a possibility of observational bias, as the assessment of adhesions was carried out by the investigator surgeons rather than by a central external assessment. Third, details of previous abdominal surgery were not known and may not have been randomized in either group. Third, although there were statistically significant reductions in operative workload and adhesion formation, the absolute reduction in operative time is relatively small. Further study will need to determine if there is a direct benefit to the patient.

## 5 CONCLUSIONS

This study showed that the use of spray‐type anti‐adhesion material was associated with a significantly lower SURG‐TLX value, lower incidence of adhesion, lower severity of adhesions, and shorter operative time. Our findings suggest that spray‐type anti‐adhesion materials may be beneficial in selected cases for the construction of a diverting ileostomy.

## AUTHOR CONTRIBUTIONS

**Emi Ota:** Conceptualization; data curation; investigation; writing – original draft; writing – review and editing. **Jun Watanabe:** Conceptualization; data curation; formal analysis; funding acquisition; investigation; methodology; project administration; supervision; writing – original draft; writing – review and editing. **Yusuke Suwa:** Conceptualization; data curation; investigation; writing – review and editing. **Masakatsu Numata:** Conceptualization; data curation; investigation; writing – review and editing. **Hirokazu Suwa:** Conceptualization; data curation; investigation; writing – review and editing. **Hiroki Ohya:** Conceptualization; data curation; investigation; methodology; writing – review and editing. **Kazuya Nakagawa:** Conceptualization; data curation; investigation; writing – review and editing. **Mayumi Ozawa:** Conceptualization; data curation; investigation; writing – review and editing. **Itaru Endo:** Conceptualization; data curation; investigation; supervision; writing – review and editing.

## FUNDING INFORMATION

This work was supported and funded by TERUMO CORPORATION, Japan.

## CONFLICT OF INTEREST STATEMENT

Jun Watanabe reports receiving honoraria for lectures from TERUMO CORPORATION, and received research funding from TERUMO CORPORATION for this article. The other authors declare no conflicts of interest. The authors Jun Watanabe and Itaru Endo are editorial members of *Annals of Gastroenterological Surgery*.

## ETHICS STATEMENT

Approval of the research protocol: This study was approved by the Institutional Review Board of Yokohama City University Medical Center (approval no. CRB3180007).

Informed consent: Written informed consent was obtained from all patients prior to enrollment.

Registry and the Registration No. of the study: This trial was registered in the Japan Registry of Clinical Trials (jRCT) as jRCTs032200155.

Animal Studies: N/A.

## Data Availability

Dr. Watanabe had full access to all data in the study and took responsibility for the integrity of the data and the accuracy of the data analysis.

## References

[1] Monk BJ , Berman ML , Montz FJ . Adhesions after extensive gynecologic surgery: clinical significance, etiology, and prevention. Am J Obstet Gynecol. 1994;170(5 Pt 1):1396–1403. 8178880 10.1016/s0002-9378(94)70170-9

[2] Menzies D , Ellis H . Intestinal obstruction from adhesions–how big is the problem? Ann R Coll Surg Engl. 1990;72(1):60–63. 2301905 PMC2499092

[3] Ellis H . The clinical significance of adhesions: focus on intestinal obstruction. Eur J Surg Suppl. 1997;(577):5–9. 9076446

[4] Scott‐Coombes DM , Vipond MN , Thompson JN . General surgeons' attitudes to the treatment and prevention of abdominal adhesions. Ann R Coll Surg Engl. 1993;75(2):123–128. 8476180 PMC2497791

[5] Okabayashi K , Ashrafian H , Zacharakis E , Hasegawa H , Kitagawa Y , Athanasiou T , et al. Adhesions after abdominal surgery: a systematic review of the incidence, distribution and severity. Surg Today. 2014;44(3):405–420. 23657643 10.1007/s00595-013-0591-8

[6] Diamond MP , Nezhat F . Adhesions after resection of ovarian endometriomas. Fertil Steril. 1993;59(4):934–935. 8458520

[7] Mettler L . Pelvic adhesions: laparoscopic approach. Ann N Y Acad Sci. 2003;997:255–268. 14644833 10.1196/annals.1290.029

[8] Duepree HJ , Senagore AJ , Delaney CP , Fazio VW . Does means of access affect the incidence of small bowel obstruction and ventral hernia after bowel resection? Laparoscopy versus laparotomy. J Am Coll Surg. 2003;197(2):177–181. 12892794 10.1016/S1072-7515(03)00232-1

[9] Sonoda T , Pandey S , Trencheva K , Lee S , Milsom J . Longterm complications of hand‐assisted versus laparoscopic colectomy. J Am Coll Surg. 2009;208(1):62–66. 19228504 10.1016/j.jamcollsurg.2008.09.003

[10] Leclercq RMFM , Van Barneveld KWY , Schreinemacher MH , Assies R , Twellaar M , Bouvy ND , et al. Postoperative abdominal adhesions and bowel obstruction. A survey among Dutch general practitioners. Eur J Gen Pract. 2015;21(3):176–182. 26161685 10.3109/13814788.2015.1055466

[11] Parker MC , Ellis H , Moran BJ , Thompson JN , Wilson MS , Menzies D , et al. Postoperative adhesions: ten‐year follow‐up of 12,584 patients undergoing lower abdominal surgery. Dis Colon Rectum. 2001;44(6):822–830. 11391142 10.1007/BF02234701

[12] Van Steensel S , Van Den Hil LCL , Schreinemacher MHF , Ten Broek RPG , Van Goor H , Bouvy ND . Adhesion awareness in 2016: an update of the national survey of surgeons. PLoS One. 2018;13(8):e0202418. 30118503 10.1371/journal.pone.0202418 PMC6097683

[13] Hackethal A , Sick C , Brueggmann D , Tchartchian G , Wallwiener M , Muenstedt K , et al. Awareness and perception of intra‐abdominal adhesions and related consequences: survey of gynaecologists in German hospitals. Eur J Obstet Gynecol Reprod Biol. 2010;150(2):180–189. 20236750 10.1016/j.ejogrb.2010.02.017

[14] Tang CL , Seow‐Choen F , Fook‐Chong S , Eu KW . Bioresorbable adhesion barrier facilitates early closure of the defunctioning ileostomy after rectal excision: a prospective, randomized trial. Dis Colon Rectum. 2003;46(9):1200–1207. 12972964 10.1007/s10350-004-6716-9

[15] Salum M , Wexner SD , Nogueras JJ , Weiss E , Koruda M , Behrens K , et al. Does sodium hyaluronate‐ and carboxymethylcellulose‐based bioresorbable membrane (Seprafilm) decrease operative time for loop ileostomy closure? Tech Coloproctol. 2006;10(3):187–190. 16969618 10.1007/s10151-006-0278-x

[16] Memon S , Heriot AG , Atkin CE , Lynch AC . Facilitated early ileostomy closure after rectal cancer surgery: a case‐matched study. Tech Coloproctol. 2012;16(4):285–290. 22618211 10.1007/s10151-012-0843-4

[17] Bertoni DM , Hammond KL , Beck DE , Hicks TC , Whitlow CB , Vargas HD , et al. Use of sodium hyaluronate/carboxymethylcellulose bioresorbable membrane in loop ileostomy construction facilitates stoma closure. Ochsner J. 2017;17(2):146–149. 28638287 PMC5472073

[18] Suto T , Watanabe M , Endo T , Komori K , Ohue M , Kanemitsu Y , et al. The primary result of prospective randomized multicenter trial of new spray‐type bio‐absorbable adhesion barrier system (TCD‐11091) against postoperative adhesion formation. J Gastrointest Surg. 2017;21(10):1683–1691. 28744742 10.1007/s11605-017-3503-1 PMC5610222

[19] Carswell CM , Claeke D , Seales WB . Assessing mental workload during laparoscopic surgery. Surg Innov. 2005;12(1):80–90. 15846451 10.1177/155335060501200112

[20] Wilson MR , Poolton JM , Malhotra N , Ngo K , Bright E , Masters RSW . Development and validation of a surgical workload measure: the surgery task load index (SURG‐TLX). World J Surg. 2011;35(9):1961–1969. 21597890 10.1007/s00268-011-1141-4 PMC3152702

[21] Ohya H , Watanabe J , Goto K , Suwa Y , Nakagawa K , Ozawa M , et al. Study protocol: a multicenter randomized controlled trial of the multifaceted workload reduction of the anti‐adhesion barrier for diverting ileostomy in laparoscopic rectal surgery, YCOG 2005 (ADBARRIER study). Int J Colorectal Dis. 2021;36(12):2763–2768. 34545454 10.1007/s00384-021-04032-3

[22] Law KE , Lowndes BR , Kelley SR , Blocker RC , Larson DW , Hallbeck MS , et al. NASA‐task load index differentiates surgical approach: opportunities for improvement in colon and Rectal surgery. Ann Surg. 2020;271(5):906–912. 30614878 10.1097/SLA.0000000000003173

[23] Kai M , Maeda K , Tasaki M , Kira S , Nakamura S , Chino N , et al. Evaluation of a spray‐type, novel dextrin hydrogel adhesion barrier under laparoscopic conditions in a porcine uterine horn adhesion model. J Minim Invasive Gynecol. 2018;25(3):447–454. 29030291 10.1016/j.jmig.2017.09.023

[24] Metwally M , Cheong Y , Li TC . A review of techniques for adhesion prevention after gynecological surgery. Curr Opin Obstet Gynecol. 2008;20(4):345–352. 18660685 10.1097/GCO.0b013e3283073a6c

[25] Cheung JPY , Tsang HHL , Cheung JJC , Yu HHY , Leung GKK , Law WL . Adjuvant therapy for the reduction of postoperative intra‐abdominal adhesion formation. Asian J Surg. 2009;32(3):180–186. 19656760 10.1016/S1015-9584(09)60392-4

[26] Kusunoki M , Ikeuchi H , Yanagi H , Noda M , Tonouchi H , Mohri Y , et al. Bioresorbable hyaluronate‐carboxymethylcellulose membrane (Seprafilm) in surgery for rectal carcinoma: a prospective randomized clinical trial. Surg Today. 2005;35(11):940–945. 16249848 10.1007/s00595-005-3061-0

[27] Cohen Z , Senagore AJ , Dayton MT , Koruda MJ , Beck DE , Wolff BG , et al. Prevention of postoperative abdominal adhesions by a novel, glycerol/sodium hyaluronate/carboxymethylcellulose‐based bioresorbable membrane: a prospective, randomized, evaluator‐blinded multicenter study. Dis Colon Rectum. 2005;48(6):1130–1139. 15868230 10.1007/s10350-004-0954-8

[28] Vrijland WW , Tseng LNL , Eijkman HJM , Hop WCJ , Jakimowicz JJ , Leguit P , et al. Fewer intraperitoneal adhesions with use of hyaluronic acid‐carboxymethylcellulose membrane: a randomized clinical trial. Ann Surg. 2002;235(2):193–199. 11807358 10.1097/00000658-200202000-00006 PMC1422414

[29] Becker JM , Dayton MT , Fazio VM , Beck DE , Stryker SJ , Wexner SD , et al. Prevention of postoperative abdominal adhesions by a sodium hyaluronate‐based bioresorbable membrane: a prospective, randomized, double‐blind multicenter study. 1996;183(4):297–306. 8843257

[30] Cezar C , Korell M , Tchartchian G , Ziegler N , Senshu K , Herrmann A , et al. How to avoid risks for patients in minimal‐access trials: avoiding complications in clinical first‐in‐human studies by example of the ADBEE study. Best Pract Res Clin Obstet Gynaecol. 2016;35:84–96. 26707194 10.1016/j.bpobgyn.2015.11.004

[31] Ten Broek RPG , Strik C , Issa Y , Bleichrodt RP , Van Goor H . Adhesiolysis‐related morbidity in abdominal surgery. Ann Surg. 2013;258(1):98–106. 23013804 10.1097/SLA.0b013e31826f4969

