# Correction to ‘A Multicenter Randomized Controlled Trial Evaluating the Effect of the Use of an Anti‐Adhesion Barrier for Diverting Ileostomy on the Multidimensional Workload in Minimally Invasive Surgery for Rectal Cancer (YCOG 2005: The ADOBARRIER Study)’

**DOI:** 10.1002/ags3.70102

**Published:** 2025-10-02

E. Ota, J. Watanabe, Y. Suwa, M, et al., “A multicenter randomized controlled trial evaluating the effect of the use of an anti‐adhesion barrier for diverting ileostomy on the multidimensional workload in minimally invasive surgery for rectal cancer (YCOG 2005: The ADOBARRIER study),” *Annals of Gastroenterological Surgery* 9, no. 5 (2025): 971–979. https://doi/full/10.1002/ags3.70009.

Errors have been identified in Tables 1 and 4 of the published article.

In Table 1:

The value for ‘Sex, Female’, column ‘AdSpray™ arm (*n* = 61)’ should be ‘23 (37.7%)’ instead of ‘38 (37.7%)’.

The value for ‘PS, 1’, column ‘Control arm (*n =* 60)’ should be ‘3 (5%)’ instead of ‘3 (5.9%)’.

In Table 4:

The value for ‘Blood loss, mL’, column ‘AdSpray™ arm (*n* = 61)’ should be ‘0 (0–5)’ instead of ‘5 (0–5)’.

The value for ‘Postoperative complications, Yes’, column ‘AdSpray™ arm (*n* = 61)’ should be ‘5 (8.2%)’ instead of ‘56 (8.2%)’.

We apologize for these errors.

